# Global Cold‐Water Coral Biodiversity Redistribution Under Projected Climate Change

**DOI:** 10.1111/gcb.70563

**Published:** 2025-10-22

**Authors:** Eliza Fragkopoulou, Lidiane P. Gouvêa, Viktória Balogh, Ester A. Serrão, Jorge Assis

**Affiliations:** ^1^ Centro de Ciências Do Mar Do Algarve (CCMAR/CIMAR LA) Campus de Gambelas, Universidade Do Algarve Faro Portugal; ^2^ Faculty of Biosciences and Aquaculture Nord University Bodo Norway

**Keywords:** biodiversity, bioregionalization, climate change, cold‐water corals, range shifts, species distribution modelling, species richness

## Abstract

Cold‐water corals (CWCs) are key ecosystem‐structuring species across the world's oceans, yet their global distribution, diversity patterns, and vulnerability to climate change remain poorly understood. Here, we delineated the global biogeography of CWCs and assessed how their biodiversity patterns may shift under future climate change scenarios. Using an ensemble of machine‐learning models, we predicted the distributions of 741 CWC species, spanning Octocoralia, Scleractinia, Antipatharia, Zoanthidae, Pennatulacea, and Filifera, under present‐day conditions and forecasted changes in species richness, community composition, and climate refugia under two contrasting Shared Socioeconomic Pathways (SSP1‐1.9 and SSP3‐7.0). Further, we identified biogeographic regions based on species co‐occurrence patterns and statistically validated them. Our results showed major biodiversity hotspots in the Gulf of Mexico and the Caribbean Sea, and delineated ten distinct bioregions, each with varying species richness, depth distribution patterns, and generally low levels of endemicity. While the global extent of the CWC biome may persist in the future, we forecasted pronounced poleward and depth shifts in species distributions, particularly under high‐emission scenarios, resulting in biodiversity losses in shallow and low‐latitude regions and increased community turnover. Our findings highlight the growing threat of climate change to CWC biodiversity and deep‐sea ecosystems and the need for urgent climate action, aligned with the Paris Agreement. By identifying biodiversity hotspots, emerging climate refugia, and regions at greatest risk, this study offers a global framework to inform conservation priorities and support efforts to safeguard CWC biodiversity in the long term.

## Graphical abstract

Cold‐water corals are key habitat providers in the world's oceans, yet their global distribution, diversity patterns, and vulnerability to climate change remain poorly understood. Our study mapped the global distribution of 741 cold‐water coral species and showed that while the overall coral biome may persist in the future, many species will shift poleward and towards deeper, colder waters, leading to losses in tropical and shallow regions. These findings identify both vulnerable hotspots and potential climate refugia to guide future conservation efforts.

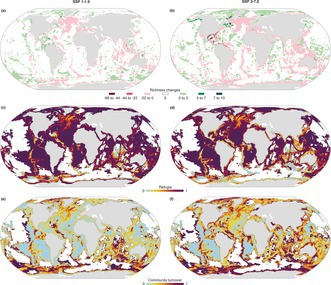

## 1 Introduction

Cold‐water corals (CWCs) are critical ecosystem‐structuring species that provide essential services across the world's oceans, from the continental shelf to abyssal depths (Cordes et al. 2017; Roberts et al. 2006). By forming complex three‐dimensional habitats, they provide feeding, breeding, and nursery grounds for a wide range of species, including many commercially important fish and invertebrates (Henry and Roberts 2017; Yesson et al. 2012). CWCs also play a key role in carbon and nutrient cycling, contributing to deep‐sea ecosystem functioning (De Clippele et al. 2021; Van Oevelen et al. 2009). Despite their ecological and economic importance, knowledge of their global diversity, distribution, and vulnerability to future climate change remains limited (Lim et al. 2020), constraining effective conservation strategies. This knowledge gap is particularly concerning given that CWCs are expected to exhibit low resilience, due to their sessile nature and slow growth rates (Hall‐Spencer et al. 2002; Huvenne et al. 2016). Most research to date has been species or region‐specific (e.g., Burgos et al. 2020; Davies and Guinotte 2011; Morato et al. 2020) or descriptive of previous data (Cairns 2007), with global biogeographical assessments lacking taxonomic resolution (e.g., Tong et al. 2023). Comprehensive biogeographic assessments, integrated with climatic predictions, are urgently needed to inform conservation planning, particularly as CWCs face mounting threats not only from climate change but also from deep‐sea mining, oil and gas extraction, and bottom‐contact fisheries.

As climate change intensifies, marine biodiversity is undergoing extensive redistributions, altering global patterns of species richness and community composition, particularly under projected high carbon emission scenarios (Assis, Fragkopoulou, et al. 2024; Pecl et al. 2017; Pinsky et al. 2020). These shifts pose major threats to ecosystem functions and services, especially when they impact keystone species such as CWCs, whose decline or complete loss can have cascading effects on the vast array of associated species (Cordes et al. 2017). CWCs are highly vulnerable to multiple anthropogenic pressures (Roberts et al. 2016), including rising sea temperatures and extreme events in the deep ocean (Fragkopoulou et al. 2023), directly affecting their ecophysiology and leading to reported population declines (e.g., Boolukos et al. 2019; Hebbeln et al. 2019). Further, cumulative and synergistic effects of deoxygenation and acidification exacerbate CWC vulnerability (Büscher et al. 2022; Hennige et al. 2020; Morato et al. 2020), highlighting the urgent need for mitigation measures. Furthermore, bottom contact fisheries and mineral extractive activities in the deep‐sea cause direct physical damage, further threatening these habitats (Hall‐Spencer et al. 2002; Huvenne et al. 2016).

Recognising their ecological importance and sensitivity to disturbance, CWCs have been designated as Vulnerable Marine Ecosystems (VMEs) under the United Nations General Assembly Resolution 61/105 (2006), which calls for the protection of fragile deep‐sea habitats from significant adverse impacts (Auster et al. 2011). This has led to the implementation of fisheries management measures, particularly in areas beyond national jurisdiction (ABNJ). However, despite this recognition and initial measures, effective conservation efforts are significantly hampered by critical knowledge gaps concerning the global distribution of CWCs, their intricate biodiversity patterns, and their capacity to withstand climate change (Henry and Roberts 2017). For instance, identifying areas of high species richness and locating potential climatic refugia—regions offering long‐term environmental stability—represents crucial information for targeted conservation. The absence of this fundamental information severely impedes the implementation of effective management and conservation strategies, including the establishment of Marine Protected Areas (MPAs) as envisioned by the post‐2020 biodiversity framework's ambitious 30% global protection target (CBD 2022).

To address the critical knowledge gaps in CWC distribution and their future vulnerability, we present a global assessment of the biogeography of 741 currently recognized CWC species (Balogh et al. 2023; Freiwald et al. 2021), representing the most extensive distribution dataset compiled to date. Using advanced machine learning species distribution models (SDMs; Peterson et al. 2011) considering biologically relevant environmental variables, we identify current patterns of biodiversity and quantify projected shifts in species richness, community turnover, and climate refugia under the latest generation of Shared Socioeconomic Pathway (SSP) scenarios of end‐of‐century climate change. We further delineate biogeographic regions based on species co‐occurrence patterns and validate their distinctiveness through multivariate analysis of similarities (ANOSIM). Our findings offer crucial insights into the biogeography and ecological responses of CWCs to climate change, informing the development of effective long‐term conservation and management strategies for vulnerable deep‐sea ecosystems worldwide.

## 2 Methods

To assess current and future patterns of CWC biodiversity, we employed species distribution modelling (Peterson et al. 2011) following established standards in biodiversity assessment protocols (Araújo et al. 2019). We used an ensemble of three high‐performance machine learning algorithms, namely Boosted Regression Trees (BRT; Elith et al. 2008), Adaptive Boosting (AdaBoost; Hofner et al. 2011), and Extreme Gradient Boosting (XGBoost; Chen and Guestrin 2016), which are well‐suited for capturing complex ecological relationships, handling low‐prevalence biodiversity datasets (Barbet‐Massin et al. 2012), and reducing overfitting through hyperparameter tuning and monotonic response constraints (Elith et al. 2008; Hofner et al. 2011).

### 2.1 Environmental and Occurrence Data

Environmental predictor variables were obtained from Bio‐ORACLE v3.0 (Assis, Fernández Bejarano, et al. 2024), covering present‐day conditions (2010–2020) and end‐of‐century climate projections (2090–2100) under two contrasting SSP scenarios: the “sustainability” SSP1‐1.9, aligned with the Paris Agreement target of reducing greenhouse emissions, and the “regional rivalry” SSP3‐7.0 of increasing emissions over time. A set of biologically relevant predictors for the benthic environment (i.e., ocean floor) was selected, including ocean temperature, salinity, dissolved oxygen, pH, primary productivity, current speed, terrain rugosity, and terrain slope (Assis, Fernández Bejarano, et al. 2024). We used the long‐term averages of minimum and maximum temperature to capture the full thermal range that defines and constrains species distributions, and the long‐term minimum for the remaining variables, as these reflect the most stressful conditions and are more likely to limit species presence.

The selection of CWC species was based on the list of cold‐water corals of UNEP (Freiwald et al. 2021), which includes species from the subclass Octocorallia (octocorals; also known as Alcyonaria) and four orders within the class Anthozoa: Scleractinia (reef‐forming corals), Antipatharia (black corals), Zoanthidae (encrusting or button polyps), and Pennatulacea (sea pens). It also includes the suborder Filifera (lace corals) within the class Hydrozoa. Within this taxonomic scope, expert‐verified occurrence distribution records for 741 taxonomically accepted CWC species were accessed from the cold‐water coral dataset, excluding those flagged as potentially erroneous (Balogh et al. 2023). Because absence data are unavailable, pseudo‐absences were generated for each species by randomly selecting points within the same realms (as defined by Costello et al. 2017) as their recorded occurrences. To minimise sampling bias and spatial autocorrelation, a spatial thinning process was applied to both occurrences and pseudo‐absences by randomly selecting one record per species within a species‐specific distance where no significant spatial autocorrelation was detected (Segurado et al. 2006). Specifically, the thinning distance was determined using correlograms evaluating Pearson's correlation coefficient of predictor variables as a function of increasing geographic distance. For species with more than 1000 occurrences, pseudo‐absences were balanced at a 1:1 ratio with presence records, while for species with fewer occurrences, pseudo‐absences were assigned using 10 model runs with a minimum of 100 per species (Barbet‐Massin et al. 2012). Additionally, to mitigate class imbalance and improve model interpretability, pseudo‐absences were segregated into clusters based on climate conditions using k‐means clustering (Senay et al. 2013).

### 2.2 Model Training, Hyperparameter Tuning, and Performance Evaluation

Model hyperparameters were optimized using the grid search method, testing all possible parameter combinations for each algorithm. For BRT, the grid search tested learning rate from 0.1 to 0.01 (step 0.001), tree complexity from 1 to 4, and number of trees from 50 to 1000 (step 50); for AdaBoost, the number of interactions varied from 50 to 250 (step 50), degrees of freedom from 1 to 12, shrinkage from 0.25 to 1 (step 0.25); and for XGBoost, gamma varied from 0 to 5, interaction depth from 1 to 4, shrinkage from 0.1 to 0.5 (step 0.1) and number of rounds from 10 to 100 (step 10). Models were trained using a 10‐fold cross‐validation framework (Roberts and Hamann 2012; Valavi et al. 2019), with independent spatial blocks defined as equal‐area hexagons matching the inferred spatial autocorrelation distances. Competitive models were trained with data distributed in nine random folds, with one fold reserved at a time for testing predictive error. Monotonicity constraints were applied to predictor variables based on their expected ecological effects on CWC distributions, an approach that strongly reduces overfitting while ensuring biologically realistic responses (Fragkopoulou et al. 2021; Hofner et al. 2011). Specifically, we assumed that maximum temperature would have a negative effect on the models' response, as increases beyond a species' tolerance cause physiological stress and mortality. In contrast, all other predictors were positively constrained, as higher values generally indicate more favourable conditions. Collinearity among predictors was assessed using the Variance Inflation Factor (VIF), with VIF > 10 indicating collinearity (Shrestha 2020). Model performance was assessed using the Boyce index, sensitivity (true positive rate), and area under the curve (AUC), with Boyce values above 0, and sensitivity and AUC above 0.5 indicating predictions better than random, and values closer to 1 indicating predictions matching observed patterns (Hirzel et al. 2006).

Per‐species ensemble distribution maps were constructed as weighted averages based on the performance of successfully converged models. Ensembles integrated predictive responses across algorithms and cross‐validation runs (Araújo and New 2007) to reduce the influence of poor fits and improve robustness. The final models, fitted with optimal combinations of hyperparameters determined by minimizing prediction error in cross‐validation, were used to assess the relative contribution of predictors and to generate partial dependency plots depicting the effect of each predictor on model responses (Elith et al. 2008).

We refined the continuous suitability maps to create biologically realistic binary presence‐absence maps using three key steps. First, we reclassified the maps using a minimum training area threshold to ensure high sensitivity (≥ 0.95; Vignali et al. 2020). Second, to prevent overprediction, which is a common issue with low‐dispersal taxa, distribution maps were clipped using a maximum dispersal constraint of 200 km (Fragkopoulou et al. 2022). This distance is a biologically informed threshold based on the average larval dispersal distance of corals (~10 days planktonic duration), estimated from a comprehensive literature review of propagule dispersal durations and biophysical modelling with daily ocean current data (Fragkopoulou et al. 2024). Specifically, we retained all continuous habitat patches that contained at least one occurrence record, while any isolated, discontinuous patches were included only if they were within 200 km of a patch with occurrences. This approach, applied both to present‐day and future projections, assumes that larvae cannot disperse across unsuitable habitat unless occurrence records demonstrate otherwise (Mendes et al. 2020). Third, both present‐day and future projections were constrained within the species' known depth distribution, based on Sea Life Base (Palomares and Pauly 2025). When species‐level depth range was not available, genus‐level averages were used (Appendix S1).

To infer species richness under present‐day and future SSP conditions, binary distribution maps for individual species were summed (i.e., stacked‐SDM; Fragkopoulou et al. 2022). Per‐pixel estimates of species gains (G) and losses (L) under future scenarios were then calculated, and community turnover was quantified as (L + G)/(SR + G), where SR refers to present‐day species richness (Thuiller et al. 2005). Per‐pixel climate refugia were also identified as the proportion of species projected to retain present‐day habitat suitability under future climate conditions, representing areas of long‐term habitat stability.

### 2.3 Bioregionalization Analysis

To delineate distinct biogeographic regions of CWCs, we employed k‐means clustering, a widely used unsupervised machine learning algorithm (Lloyd 1982). This method partitioned the global ocean grid cells into clusters (here representing bioregions) based on the similarity of their predicted CWC species distributions and co‐occurrence patterns. Each grid cell was represented by a vector of predicted occurrence probabilities (i.e., a continuous variable ranging between 0 and 1) for each of the 741 CWC species in our dataset. The k‐means algorithm iteratively assigns grid cells to the nearest bioregion centroid, minimizing the within‐cluster sum of squares (WCSS) and effectively clustering cells with similar species assemblages. The optimal number of bioregions (*k*) was determined by systematically testing values ranging from 2 to 30 and selecting the solution that maximized the ratio of between‐cluster sum of squares (BCSS) to within‐cluster sum of squares (WCSS; Zhao et al. 2009). A higher BCSS/WCSS ratio indicates more distinct and ecologically meaningful bioregions (Steinley and Brusco 2011). To further explore hierarchical relationships among the identified bioregions, we performed hierarchical clustering on the final *k*‐means centroids using the Ward.D2 linkage method (Ward 1963). The resulting dendrogram illustrated biogeographic affinities and divisions among bioregions based on shared species assemblages. Finally, for each bioregion, we estimated species richness and endemism (i.e., species found exclusively in a single bioregion), along with their depth distributions using high‐resolution bathymetric data from the General Bathymetric Chart of the Oceans (15 arc sec, ~450 m; GEBCO 2023). Changes in species richness and community turnover under climate change were also estimated at the bioregion level, following the per‐pixel methods described above.

To assess the statistical significance of species composition differences among bioregions, we performed an Analysis of Similarities (ANOSIM) using the Bray–Curtis dissimilarity metric (Clarke 1993). ANOSIM is a non‐parametric test that evaluates whether community composition varies significantly among predefined groups (i.e., bioregions). Given the large size of our dataset (7,923,094 grid cells × 741 species), we implemented a randomized subsampling approach to conduct ANOSIM efficiently. Specifically, we performed 100 independent ANOSIM runs, each based on a random selection of 10,000 grid cells from the full dataset, ensuring an adequate representation of species composition across bioregions while maintaining computational feasibility. The *R*‐statistic and *p*‐value of each interaction were recorded, and the mean *R*‐statistic and the proportion of significant runs (*p* < 0.05) were estimated.

All analyses were conducted in R (R Development Core Team 2025). The datasets, SDM code, and resulting distribution layers (per species, 0.05° resolution) are openly available in permanent repositories (see Data Availability Statement).

## 3 Results

**FIGURE 1:**
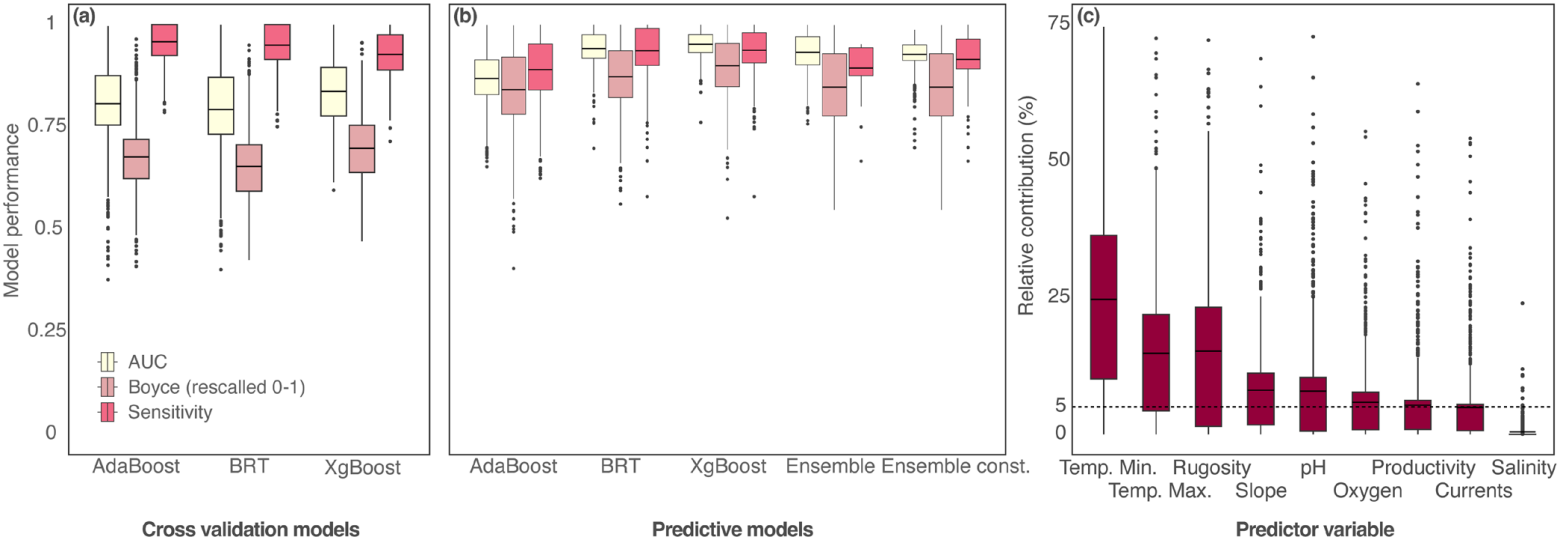
Species distribution model performance, measured as AUC, Boyce, and Sensitivity, shown for (a) cross‐validation and (b) final predictive models across Adaptive Boosting (AdaBoost), Boosted Regression Trees (BRT), and Extreme Gradient Boosting (XGBoost) algorithms and their ensemble predictions without and with dispersal constraints (Const.). (c) Relative contribution of predictor variables to the ensemble models. Detailed per‐species results are provided in Appendix S2. Boyce index values were rescaled to 0–1 for consistency with other performance metrics.

The models developed for the 741 CWC species (Appendix S1) demonstrated high performance in both cross‐validation (average AUC 0.81 ± 0.10, Boyce 0.35 ± 0.18, sensitivity 0.94 ± 0.05) and final predictions (AUC 0.92 ± 0.05, Boyce 0.69 ± 0.19, sensitivity 0.92 ± 0.06) across the three machine learning algorithms used (Appendix S2). Model performance increased when ensembling the algorithms into a unique consensus and accounting for dispersal constraints to suitable reachable areas (average AUC 0.93 ± 0.04, Boyce 0.69 ± 0.19, sensitivity 0.92 ± 0.07, Figure 1; Appendix S2). Overall, model uncertainty was low, with the maximum standard deviation in probability of occurrence remaining below 0.03 (potential range between 0 and 1) across algorithms (Figure S1).

Predictive models identified minimum (24.7%) and maximum (14.8%) temperature and terrain rugosity (15.2%) as the primary drivers of the CWC biome distribution, collectively contributing an average of 54.8% to model performance (Figure 1; Appendix S3). Slope, pH, oxygen, productivity, and current speed had moderate average contributions, ranging from 8.1% to 4.9%, while salinity had the lowest contribution at 0.4% (Figure 1; Appendix S3). For specific species, certain environmental variables played dominant roles in determining distributions (Appendix S3). Collinearity was detected between maximum and minimum temperatures and between terrain slope and rugosity in over 80% of the models (Table S1). However, all predictors were retained, as maximum and minimum temperatures capture distinct ecological limits and were modelled with opposite monotonic constraints, and slope and rugosity represent different ecological processes (e.g., particle deposition and food uptake versus habitat complexity and availability).

**FIGURE 2:**
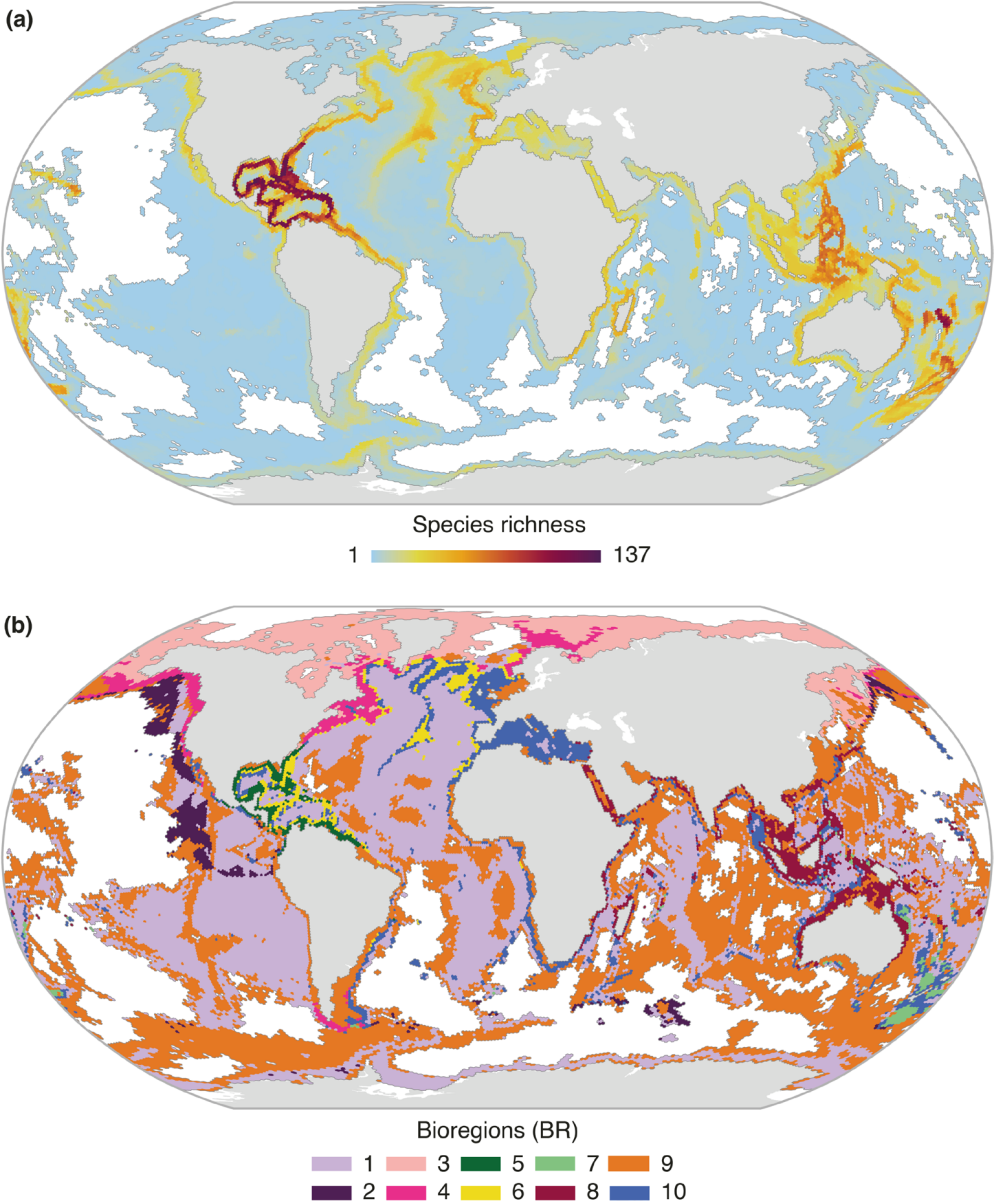
(a) Present‐day species richness distribution for 741 species of cold‐water corals. (b) Cold‐water coral bioregions (BR) derived from species distributions. Areas with no species present are shown in white.

Stacking individual distribution models for present‐day conditions predicted a total suitable geographic area of 179,905,947 km^2^, distributed at an average depth of 456 m (range from 0 to 7377 m; Appendix S4). Depth estimates represent averages within 0.05° grid cells and may include coastal cells at 0 m. Species richness patterns were not evenly distributed across oceans, with the highest species richness (up to 137 species) predicted in the Western Atlantic, from North Carolina, USA to French Guiana, including the Caribbean Islands (Figure 2a). Additional richness hotspots were predicted in the Western Pacific, spanning from Japan to New Zealand, with the highest richness in New Caledonia (Figure 2a). Moderate species richness was predicted in regions such as Eastern Africa (including Madagascar), the Northeastern Atlantic (including the Mediterranean Sea), and the North Atlantic Ridge (Figure 2a). Conversely, low CWC richness (i.e., poor spots) was mostly predicted in deep offshore areas (Figure 2a).

**FIGURE 3:**
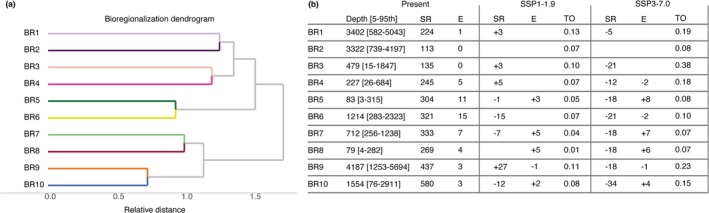
(a) Bioregionalization dendrogram showing relative distance among the identified bioregions (BR). (b) Average depth [5–95th quantile], species richness (SR), and endemicity (E) for present‐day conditions, their respective projected changes, and community turnover (TO) under SSP1‐1.9 and SSP3‐7.0 end‐of‐century climate change scenarios. Turnover ranges from 0 (no change in species composition) to 1 (complete replacement).

The bioregionalization analysis, based on the distribution of CWC species, identified 10 distinct bioregions (BR; numbered as depicted in Figure 2b). These bioregions varied greatly in geographic extent, with some, such as BR1 and BR9, exhibiting a global distribution, while others were more restricted, such as BR2 confined to the Southeastern Pacific and BR5 to the Gulf of Mexico and the Caribbean Sea (Figure 2b). Species composition varied among bioregions across the different taxonomic levels, with Octocorallia class contributing the most (58.76%) to defining the assemblages (higher variability among bioregions), and Malacalcyonacea (43.12%), Scleralcyonacea (29.40%), and Scleractinia (21.61%) at the order level (Appendices S5 and S6). At the family level, *Primnoidae* contributed the most to bioregional differentiation (29.55%), followed by *Stylasteridae* (8.99%) and *Acroporidae* (8.61%; Appendix S7). In terms of species richness, BR10 exhibited the highest richness (580 species), primarily distributed across the Mediterranean Sea and global continental shelf regions, whereas BR2 had the lowest richness (113 species), mainly distributed in offshore areas of the Eastern Pacific (Figures 2b and 3). Endemicity (species found exclusively in a single bioregion) was highest in BR6 and BR5 (15 and 11 species, respectively) despite their relatively restricted geographic distribution in the North Atlantic. In contrast, BR1 harboured only one endemic species (*Solumbellula monocephalus*), while BR2 and BR3 had no endemic species (Figure 3; Appendix S8). The generally low endemicity levels found arise from species being typically distributed across three bioregions (Figure S2). Depth distribution patterns varied widely among bioregions, with average depths ranging from 79 m in BR8 to 4187 m in BR9, highlighting distinct environmental and depth preferences (Figures 3, S3–S12). The ANOSIM analysis yielded highly consistent results across 100 independent iterations, with an average *R* = 0.50, indicating differentiation among bioregions, yet with some species overlap. The identified bioregions differed (*p* = 0.01) across all iterations, confirming that they represent distinct species assemblages (Appendix S9).

**FIGURE 4:**
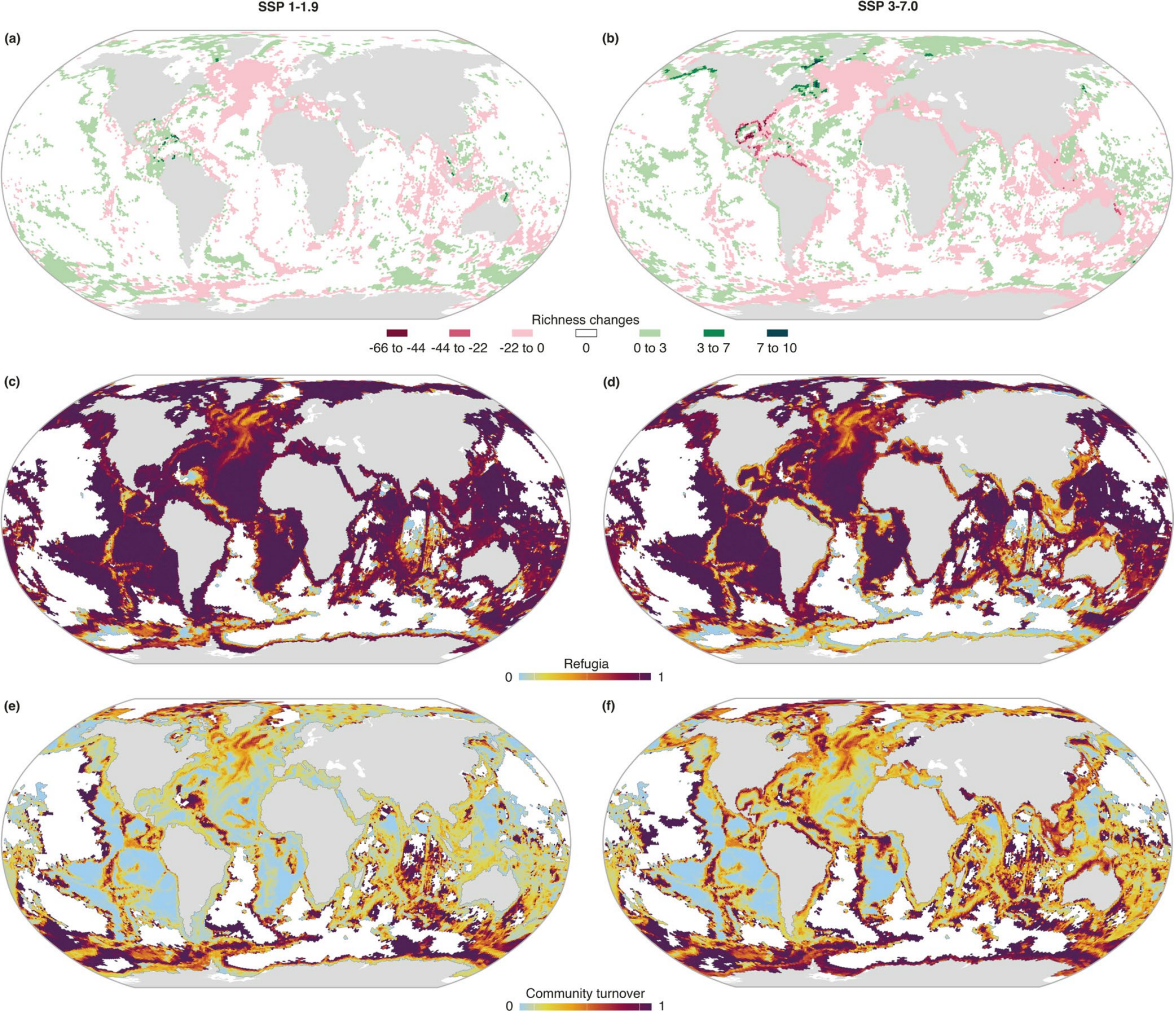
Projected end‐of‐century changes under SSP1‐1.9 and SSP3‐7.0 in (a, b) species richness, (c, d) climate refugia, and (e, f) community turnover of cold‐water corals. Climate refugia represent the fraction of species predicted to retain suitable habitat (0 = none persist; 1 = all persist). Turnover ranges from 0 (no change in species composition) to 1 (complete replacement). Areas with no species present are shown in white.

Future projections under contrasting SSP scenarios estimated minimal net changes in the total global extent of the CWC biome, with suitable areas increasing by 1.6% under SSP1‐1.9 and decreasing by 0.4% under SSP3‐7.0 (Appendix S10). However, at the species level, pronounced poleward and depth shifts were projected, particularly under the high‐emission scenario SSP3‐7.0. The average depth distribution is projected to shoal by 6.7 m under SSP1‐1.9 and to deepen by 26.0 m under SSP3‐7.0 (Appendix S4). Extensive changes in species richness are projected globally, with the greatest gains (up to 10 species) estimated mostly in deeper offshore areas and the Arctic, particularly under SSP3‐7.0 (Figure 4). Conversely, species richness losses are expected to be disproportionately larger, primarily affecting the global coastal regions and the northern Mid‐Atlantic Ridge. The most severe losses, up to 66 species, are projected in the Central Western Atlantic (Figure 4).

Despite regional losses, broad climatic refugia—areas where habitat suitability is projected to persist—were identified globally under both scenarios, covering 93.3% of present‐day distribution under SSP1‐1.9 and 89.2% under SSP3‐7.0 (Figure 4). Areas of limited refugia were mostly identified in the southern hemisphere, including offshore regions of the Indian and Southern Oceans, particularly across the Antarctic coastline (Figure 4). However, even within refugial areas, extensive shifts in community composition were projected, particularly in the deep offshore regions and under the high‐emission scenario SSP3‐7.0 (Figure 4). At the bioregional level, species richness declined moderately under SSP1‐1.9, with losses ranging from 1 to 15 species (Figure 3). In contrast, BR9 was projected to have the greatest gains in richness (+27 species), followed by BR4 (+5 species). Despite these changes, community turnover remained relatively low, reaching 0.13 in BR1 (Figure 3). Under SSP3‐7.0, species richness declined across all bioregions, with BR10 experiencing the highest loss (34 species), while turnover increased, reaching 0.38 in BR3 (Figure 3). Endemism generally increased under both scenarios (Figure 3), suggesting a potential contraction in species distribution ranges.

## 4 Discussion

Our study provides a global perspective on CWC biodiversity, mapping species richness and biogeographic structure, and their trajectories under climate change, substantially increasing the taxonomic and geographic resolution of previous approaches (e.g., Burgos et al. 2020; Morato et al. 2020; Tong et al. 2023). While suitable CWC habitat is projected to persist under end‐of‐century climate change, species‐level redistributions, mainly poleward and depth shifts, will drive considerable community turnover at local and regional scales, with the magnitude of change strongly tied to future emission trajectories.

Our machine learning ensemble, trained with expert‐verified data and ecologically relevant environmental variables, achieved high performance and low uncertainty in predicting CWC biogeographical patterns. Integrating dispersal constraints reduced overprediction without requiring prior knowledge of species dispersal ecology (Cooper and Soberón 2018; Mendes et al. 2020), a major advantage in marine systems where dispersal dynamics remain poorly understood (Álvarez‐Noriega et al. 2020). At global scales, CWC distribution was primarily shaped by physiological constraints (minimum and maximum temperature) and habitat complexity (terrain rugosity). Temperature reflects the stenothermal tolerances of many CWC species (e.g., Buhl‐Mortensen et al. 2015; Davies and Guinotte 2011), while terrain rugosity captures small‐scale habitat heterogeneity (providing suitable substrates) and hydrodynamic conditions for coral attachment and feeding (e.g., Cordes et al. 2017; Davies and Guinotte 2011; Roberts and Cairns 2014). High rugosity also reduces sediment accumulation and increases turbulence, promoting larval settlement (e.g., Burgos et al. 2020; Chimienti et al. 2019). Although some predictors were collinear, boosting algorithms are generally robust to correlation, particularly when monotonic constraints allow opposite trends, as with minimum and maximum temperature (Chowdhury et al. 2021; Hofner et al. 2011). In contrast, collinearity between slope and rugosity makes their contributions harder to disentangle, as both may reflect overlapping processes. Specific‐species drivers such as oxygen and pH variability further align with previous experimental evidence of CWC sensitivity to hypoxia and ocean acidification (e.g., Morato et al. 2020; Puerta et al. 2020; Roberts et al. 2016).

By stacking per‐species models, we identified geographic centers of species richness and projected climate‐driven shifts. Richness peaked in the Western Atlantic and Indo‐Pacific coastal and continental shelf areas, while offshore regions were less diverse. These diversity centers align with those proposed in earlier global estimates (Cairns 2007), although with regional differences, likely due to the species pools and methods considered. The bioregionalization analysis identified 10 distinct bioregions, with generally low endemicity as species typically spanned three bioregions. Exceptions include BR5 and BR6 in the Gulf of Mexico and Caribbean Sea, which had the highest number of endemic species and coincided with the main CWC richness hotspot. Bioregionalization was primarily driven by species compositions and taxonomic turnover, rather than by endemicity patterns strictly. *Primnoidae*, the most diverse family in our dataset (100 species followed by 52 in *Stylasteridae*), contributed strongly to the taxonomic turnover across bioregions due to its broad ecological and geographic distribution (Cairns and Bayer 2009). Furthermore, bioregions were structured more by depth than latitude, ranging from shallow coastal communities (e.g., BR8) to deep‐sea environments (e.g., BR9), highlighting the influence of depth‐related environmental gradients in shaping distributions (e.g., Arantes et al. 2009; Braga‐Henriques et al. 2013) and aligning with known drivers of coral diversification (Campoy et al. 2023).

Our results reinforce global marine biodiversity trends (e.g., Assis, Fragkopoulou, et al. 2024; Pecl et al. 2017; Pinsky et al. 2020) and extend evidence on CWC responses (e.g., Gasbarro et al. 2022; Morato et al. 2020), showing that climate change, particularly under the high‐emission scenario (SSP3‐7.0), will drive poleward and depth range shifts, with habitat contractions at lower latitudes and shallower areas. Species richness gains, driven by range expansions, are primarily projected in the northern hemisphere, especially across Alaska, Newfoundland, and the Canadian Arctic. Fossil evidence supports CWCs' capacity for broad climate‐induced redistributions (e.g., Boavida et al. 2019; De Carvalho Ferreira et al. 2022), but rapid climate change (Brito‐Morales et al. 2020), anthropogenic impacts (Hall‐Spencer et al. 2002; Huvenne et al. 2016), and their slow growth rates and limited dispersal may constrain the extent of the projected shifts. In deeper offshore environments, increases in species richness are anticipated as CWC distributions deepen by an average of 26 m, aligning with previous research (e.g., Morato et al. 2020). However, at lower latitudes, depth shifts might only partially mitigate habitat loss, as extensive species declines are projected globally across shallow coastal regions, particularly in the Gulf of Mexico. In the southern hemisphere, modest species richness gains are expected, occurring primarily in the southern Pacific under low emissions, while richness losses dominate under SSP3‐7.0.

At the biogeographical level, low‐emissions drive localized richness gains (e.g., BR9 and BR4) and relatively low community turnover, whereas high‐emissions cause widespread losses, turnover, and higher endemism due to range contractions. Although broad potential climate refugia were identified globally, their effectiveness in safeguarding CWC diversity may be limited by shifts in community structure and species interactions, a concern particularly relevant for offshore regions of the Indian and Southern Oceans, including the Antarctic coastline, where refugia potential is most restricted.

The projected shifts are likely to have profound impacts on community structure, particularly under increased emissions. Modest richness gains under SSP1‐1.9 may turn into widespread losses under SSP3‐7.0, increasing species turnover, both at the local and regional level. Local extinctions, invasions, and range shifts could reshape ecosystem composition (e.g., Boolukos et al. 2019; Gasbarro et al. 2019), particularly in deep offshore regions, the Arctic, and the Antarctic, where the most extensive changes are expected. While some areas may experience transient increases in species richness, these gains could reflect invasions by opportunistic species rather than a net increase in biodiversity (Boolukos et al. 2019). Meanwhile, the increase in endemism suggests that species with shrinking distributions may be more vulnerable to additional environmental and anthropogenic stressors (Danovaro et al. 2020). These compositional changes could disrupt key ecological functions, including habitat provisioning, nutrient cycling, and trophic dynamics (Huvenne et al. 2016), while the loss of keystone CWC species reduces habitat complexity, threatening dependent fauna (Boolukos et al. 2019; Puerta et al. 2020). However, uncertainties remain due to limited understanding of CWCs' ecological roles and interactions, warranting further research (Cordes et al. 2017).

Despite strong model performance, several limitations in species distribution modelling and climate change projections merit consideration. First, occurrence records may be biased by uneven sampling and limited taxonomic resolution, particularly in the deep sea, potentially underrepresenting certain areas and species (Hughes et al. 2021). Second, while incorporating dispersal constraints is crucial for sessile organisms like CWCs (Fragkopoulou et al. 2022), using a fixed dispersal distance may oversimplify the varying CWC dispersal potentials, influenced by life history traits and oceanographic conditions (Álvarez‐Noriega et al. 2020; Legrand et al. 2024), leading to potential discrepancies between projected and realised distributions (Assis, Legrand, et al. 2024). Another limitation is the exclusion of biotic interactions, such as habitat competition (Pinsky et al. 2020), which can shape CWC distributions at finer spatial scales but are rarely included in SDMs due to global data gaps. Similarly, our models omit crucial factors like hard substrate availability, for which comprehensive global datasets are lacking. Lastly, uncertainties in SSP climate scenarios, arising from climate model physics and the range of emission pathways, can inevitably propagate into our projected distributions. As a result, our models may be overestimating CWC distributions, particularly in regions where oceanographic barriers, species interactions, substrate availability, and SSP uncertainty impose additional distribution constraints.

Our findings highlight the vulnerability of CWCs to future climate change, particularly under high‐emission scenarios, with projected shifts in richness and community composition highlighting the urgency of mitigation aligned with the Paris Agreement. Potential expansions into sensitive Arctic regions and biodiversity losses in the already stressed low‐latitude and coastal habitats emphasize the need for ambitious emission reductions and targeted conservation. By identifying biodiversity hotspots and future environmental stability areas, our study provides actionable guidance for MPA designation, supporting the post‐2020 global biodiversity framework (CBD 2022) and informing management of Vulnerable Marine Ecosystems, including seamounts, hydrothermal vents, and sponge fields (Auster et al. 2011). Protecting biodiverse and resilient regions is also critical for addressing anthropogenic pressures such as bottom trawling and deep‐sea mining. For instance, the overlap between major oil and gas extraction zones in the Gulf of Mexico and the global CWC biodiversity hotspot amplifies ecosystem risks (Murawski et al. 2020). Finally, our bioregionalization reveals distinct assemblages shaped by richness, endemicity, and depth preferences, enabling global comparisons and spatially explicit conservation strategies. To support long‐term protection of these vital and vulnerable deep‐sea ecosystems, we provide comprehensive, high‐resolution species‐level projections (Gouvêa et al. 2024) under FAIR data principles (Findable, Accessible, Interoperable, and Reusable), laying a foundation for future research and collaborative conservation efforts.

## Author Contributions

**Eliza Fragkopoulou:** conceptualization, data curation, formal analysis, methodology, writing – original draft, writing – review and editing. **Lidiane P. Gouvêa:** conceptualization, writing – review and editing. **Viktória Balogh:** conceptualization, data curation, writing – review and editing. **Ester A. Serrão:** writing – review and editing. **Jorge Assis:** conceptualization, data curation, formal analysis, funding acquisition, supervision, writing – original draft, writing – review and editing.

## Conflicts of Interest

The authors declare no conflicts of interest.

## Supporting information


**Data S1:** gcb70563‐sup‐0001‐DataS1.zip.

## Data Availability

All data used in this study are openly available, and their sources have been cited in the methods. The R code used in the analyses, along with per‐species VIF values, can be accessed at https://doi.org/10.6084/m9.figshare.29086343. Per‐species predictive range maps are available at https://doi.org/10.6084/m9.figshare.23749179.
