# Supplementary material for: Global Cold‐Water Coral Biodiversity Redistribution Under Projected Climate Change
Source: Glob Chang Biol. 2025 Oct 22;31(10):e70563. doi: 10.1111/gcb.70563 (PMC12541565; doi:10.1111/gcb.70563)
Supplement: Supplementary file 1 — Data S1: gcb70563‐sup‐0001‐DataS1.zip. [file GCB-31-e70563-s001.zip › supinfo/Supplemetary Information.docx]

**Global cold-water coral biodiversity patterns under climate change**

**Supplementary information**

Table S1. Mean Variance Inflation Factor (VIF) per predictor variable across all species models, and percentage of models in which VIF exceeded 10 (threshold indicating multicollinearity of predictors). Per species VIF values are available in the Figshare repository (see Data Availability Statement).

| **Variable** | **Mean VIF** | **VIF > 10 (%)** |
| --- | --- | --- |
| Temperature maximum | 54.9 | 82.5 |
| Temperature minimum | 49.7 | 74.8 |
| Terrain rugosity | 17.5 | 79.5 |
| Terrain slope | 17.4 | 78.4 |
| Dissolved oxygen | 5.17 | 10.8 |
| pH | 4.70 | 8.37 |
| Primary productivity | 3.11 | 0.27 |
| Current speed | 1.20 | 0 |
| Salinity | 1.83 | 0 |

**
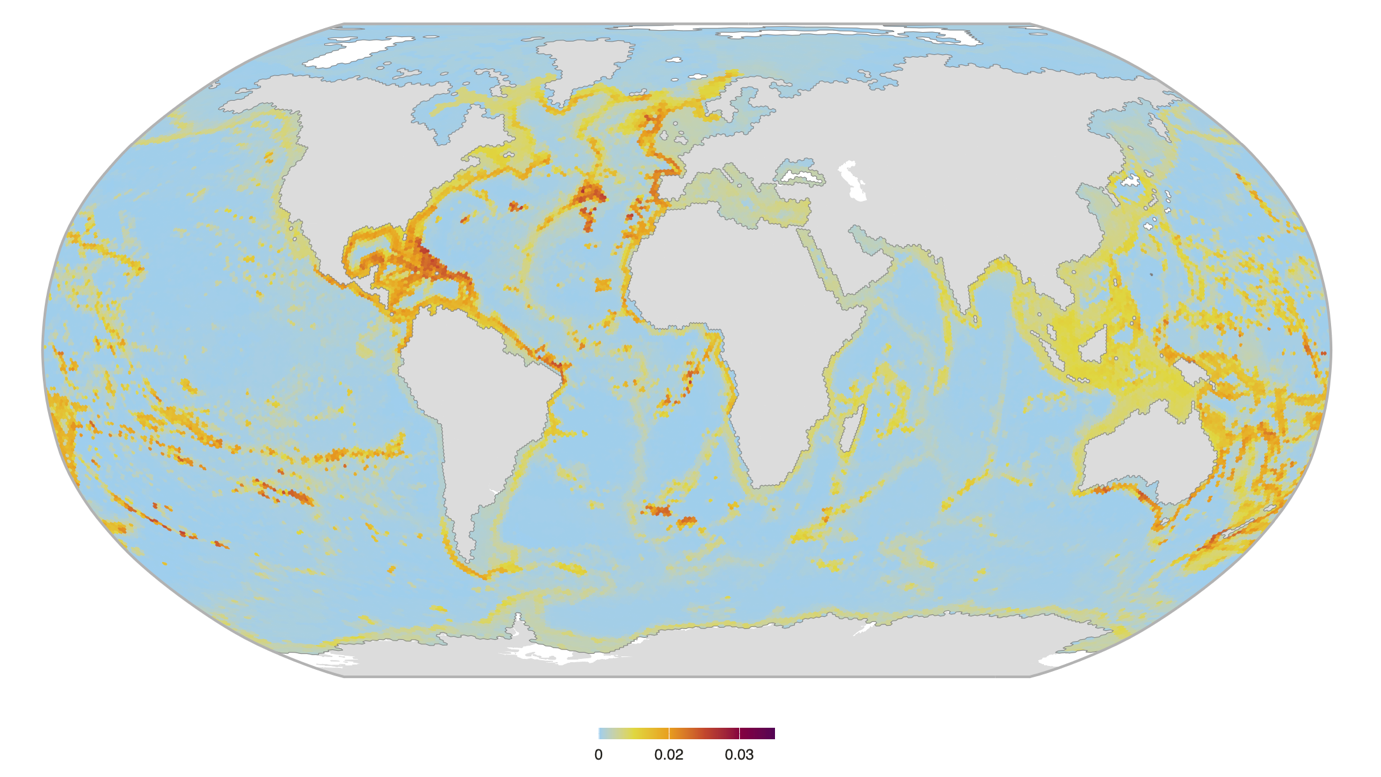
**

Figure S1. Species distribution model uncertainty for present-day conditions based on the standard deviation of probability of occurrence, as predicted across algorithms and cross-validation rounds [range 0-0.033].

Figure S2. Histogram showing how many bioregions each cold-water coral species occupies.

Figure S3. Depth distribution in bioregion (BR) 1.

FigureS4. Depth distribution in bioregion (BR) 2.

FigureS5. Depth distribution in bioregion (BR) 3.

FigureS6. Depth distribution in bioregion (BR) 4.

Figure S7. Depth distribution in bioregion (BR) 5.

Figure S8. Depth distribution in bioregion (BR) 6.

Figure S9. Depth distribution in bioregion (BR) 7.

Figure S10. Depth distribution in bioregion (BR) 8.

Figure S11. Depth distribution in bioregion (BR) 9.

Figure S12. Depth distribution in bioregion (BR) 10.

Appendix S1 - List of modelled species, number of records, known depth range and taxonomical information.

Appendix S2 - Per species model performance

Appendix S3 - Per species environmental variable contributions

Appendix S4 - Per species modelled depth distributions

Appendix S5 - Class relative contribution to bioregions

Appendix S6 - Order relative contribution to bioregions

Appendix S7 - Family relative contribution to bioregions

Appendix S8 - List of endemic species for present-day conditions

Appendix S9 - Summary of multivariate analysis of similarities (ANOSIM)

Appendix S10 - Changes in biome suitable area under climate change
